# Untreated hypertension in the UK household population — Who are missed by the general health checks?

**DOI:** 10.1016/j.pmedr.2016.05.007

**Published:** 2016-05-17

**Authors:** Jakob Petersen, Michaela Benzeval

**Affiliations:** Institute for Social and Economic Research, University of Essex, Colchester CO4 3SQ, United Kingdom

**Keywords:** Hypertension, Cardiovascular diseases, Demographic and health surveys, Screening, Primary prevention

## Abstract

Hypertension is an age-related, long-term condition and a leading risk factor for premature death and disability worldwide. Due to its asymptomatic nature it can often be left undiagnosed. Long-term treatment is available, but blood pressure can also be reduced through health behaviour changes in weight control, smoking cessation, higher physical activity levels, reduced salt and alcohol intake, and healthful diets if discovered early. This paper investigates the prevalence and characteristics of those with untreated (compared to treated) hypertension who did not have a history of cardiovascular disease (CVD); a group who is in effect missed by general health checks.

Untreated hypertension was studied in 8933 individuals aged 40–74 years representative of the UK household population, who were interviewed and underwent a physical health examination in their home, 2010–2012. The prevalence of untreated hypertension without a history of CVD was 7% for men, 2% for women, and 5% overall. Untreated hypertension was particularly high among the 55–64 year age group.

Age and sex-adjusted analyses found strong positive associations with male gender, smoking, self-reported good–excellent health, full fat dairy preference, white bread preference, higher alcohol consumption, and living alone. Strong negative associations were found for possessing 5 + prescription drugs, statins or antiplatelets, being diagnosed with diabetes or possessing antidiabetics, and long-term limiting illness status.

Notably, many reported their health as good to excellent. A fact which emphasises the importance of motivating individuals to take part in the general health checks for an asymptomatic condition such as hypertension.

## Highlights

• Prevalence of untreated hypertension without CVD among 40-74 year-olds was 5%
• Untreated hypertension was strongly associated with males, smoking, poor diet, and living alone
• Most with untreated hypertension reported their health as good to excellent

## 1 Introduction

Hypertension is a common, asymptomatic, age-related, long-term condition reported as the leading risk factor for premature death and disability in the 2010 Global Burden of Disease report (Lim et al., 2012). At ages 40–69 years, each difference of 20 mm Hg in systolic blood pressure is associated with more than a twofold difference in the stroke death rate, and with twofold differences in the death rates from ischaemic heart disease and other vascular causes (Lewington et al., 2002). Hypertension is also one of the main risk factors addressed in the English government's recently published strategy for combating cardiovascular disease (CVD) (Department of Health, 2013). Due to the fact that it is an asymptomatic condition that can only be diagnosed based on blood pressure measurements it can often be left undiagnosed. Once diagnosed hypertension can be controlled through long-term use of antihypertensive medication. Blood pressure can also be reduced and managed through health behaviour changes in weight control, smoking cessation, higher physical activity levels, reduced salt and alcohol intake, and healthful diets if discovered early (Department of Health, 2013, Yang et al., 2012).

The English government set out the new CVD outcome strategy in 2013 to improve CVD prevention, treatment pathways, and long-term care (Department of Health, 2013). Central to the strategy for primary prevention, and a major investment, is the NHS Health Check screening programme offering free CVD risk assessments to 40–74 year olds not already diagnosed with CVD, diabetes, or renal disease. The programme was rolled out in its first phase 2009–2012 and continues to be implemented nationally under a new 5-year plan from 2013. The evidence base for the general health checks programme was models showing that the increase in early diagnosis of asymptomatic, high CVD-risk, yet treatable conditions such as hypertension, diabetes and dyslipidaemia together with directed lifestyle advice would deliver cost-effectiveness and longer, healthier lives (Department of Health, 2013). Uptake has so far been close to 50% (Artac et al., 2013b). General practice audits suggest that uptake has been greater among older age groups and higher in non-smokers than smokers (Artac et al., 2013a, Artac et al., 2013b, Cochrane et al., 2013, Dryden et al., 2012, Kumar et al., 2011).

The overall aim of this study was to identify the characteristics of individuals with untreated (compared to treated) hypertension who did not have a history of CVD in the general population in order to understand who in effect are not reached by the NHS Health Check programme. Thus, we calculated the prevalence of untreated CVD-free hypertension in the UK household population. Subsequently we described the health, health behaviours, social and economic characteristics of individuals with untreated versus treated CVD-free hypertension. We used data from *Understanding Society*, a general household survey, which covers a broad section of the population and not only those in frequent contact with the healthcare system. It is in that way possible to capture individuals who are in effect hard to reach by general screening programmes.

## 2 Methods

### 2.1 Data source

This study was based on data from *Understanding Society*, a general longitudinal household survey initiated in 2009 (Wave 1) (University of Essex, 2014), and included data from a home health assessment visit in May 2010–July 2012 (carried out an average 148 days (SD 26) after the Wave 2 interview). *Understanding Society* was designed as a stratified, clustered, equal probability sample study (Lynn, 2009). The household response rates at baseline (Wave 1) were 57% and 87% of adults within these households took part. At Wave 2 72% of eligible adults took part, and of those interviewed in English, resident in Great Britain, excluding pregnant women, 59% took part (Lynn and Knies, 2016, McFall et al., 2014).

### 2.2 Identification of individuals eligible of antihypertensive therapy

The health assessment interview was conducted by a nurse in the respondent's own homes and included a short questionnaire, a range of physical measurements, and the nurse coding the medications in the respondent's possession (National Centre for Social Research, 2010). Blood pressure was measured three times with the Omron HEM 907; respondents were asked to sit quietly for five minutes before the measurements were taken using the right arm where possible. Only the second and third measurements were used here to avoid the ‘white coat’ effect, i.e. the fact the blood pressure may become raised initially in apprehensive individuals. Untreated hypertension was defined as individuals without any history of CVD, who were eligible to antihypertensive treatment and did not possess any antihypertensives. Eligibility for antihypertensive therapy followed NICE guidelines, i.e. individuals with stage 2 hypertension (150 mm Hg systolic/95 mm Hg diastolic home measurement; average of second and third measurements) and individuals with stage 1 (135/85) with either diabetes or a 10 year CVD risk exceeding 20% (NICE, 2011). Anti-hypertension therapy was defined as anti-hypertensive medication including diuretics. These are listed in British National Formulary (BNF) (BMJ/RPS, 2009) sections 2.2.1–2.2.8, beta blockers (BNF 2.4), ACE inhibitors (BNF 2.5.5.1, 2.5.5.3), calcium blockers (BNF 2.6.2), and other drugs affecting blood pressure (BNF 2.5.1–2.5.4).

### 2.3 Variables in the analysis

To understand who might be missing general health checks for diagnosis and treatment of hypertension, a range of variables were investigated including risk factors for CVD (Department of Health, 2013) and non-attendance in participatory health check programmes (Dryden et al., 2012). Variables included were age (40–54 years, 55–64 years, 65–74 years), gender, living alone, education (higher education, GCSE/A-level, none), net equivalised monthly household income, current cigarette smoking, participation in the recommended 150 min of moderate intensity physical activity per week (NICE, 2013) measured as participation in moderate intensity sports (up to 180 min per week assuming one hour sessions), brisk or fast paced walking up to 40 min per day, and cycling to work in time taken to commute (no/yes), 5 + portions of fruit/vegetables per day (no/yes), white bread preference (no/yes), full fat dairy preference (no/yes), long-term limiting illness (LTLI) (no/yes), diabetes diagnosis or medication (no/yes), general health considered good–excellent (no/yes), alcohol consumption on heaviest day of drinking in the previous week relative to the recommended 3 daily units for men and 2 for women (NHS, 2014) (< 1.5 times recommended levels, 1.5 +, no self-completion data available), obesity defined by measured height and weight (BMI 30 +: no/yes), possession of 5 + prescription drugs (no/yes), statins (no/yes), antiplatelets (no/yes), antidepressants (no/yes), Townsend area deprivation quintile at Census 2011 lower layer super output area-level (Townsend, 1987), and rural locality (Bibby and Brindley, 2013). Longterm limiting illness information was based on the survey question, “Does [name] have any long-standing physical or mental impairment, illness or disability? By ‘long-standing’ I mean anything that has troubled [name] over a period of at least 12 months or that is likely to trouble [name] over a period of at least 12 months”. The Townsend score is a composite area deprivation index with four dimensions: unemployment, lack of home ownership, lack of car ownership, and overcrowding (Townsend, 1987).

### 2.4 Data analysis

CVD risk was measured using desk-based Framingham/Joint British Societies-2 equations (D'Agostino et al., 2008, JBS, 2005). Descriptive statistics were produced for CVD-free untreated hypertension versus CVD-free treated hypertension. Multivariable logistic regression for complex survey design (StataCorp, 2013) was used to describe the risk factors for untreated (compared to treated) hypertension in 40–74 year olds without a history of CVD. All results were weighted cross-sectionally to correct for unequal selection probabilities, differential non-response, and sampling error so representative of the UK population (Knies, 2014, Lynn et al., 2012).

### 2.5 Ethics

Ethical approval for the Understanding Society study was obtained from the National Research Ethics Service Oxfordshire and the Ethics Committee of the University of Essex.

## 3 Results

A total of 9644 individuals fulfilled the 40–74 year age criterion for the study. Of these, 711 (7%) did not have data for height, weight, three blood pressure measurements, or lacked medical history about CVD status and were excluded from the analyses. The analyses were based on the remaining 8933 individuals (7684 in weighted analysis, Table 1). The majority identified themselves as British/English/Scottish/Welsh and only 8% identified themselves as belonging to another ethnic group (mainly Any other White, Indian, Irish, or Caribbean).

Of the 8933 participants in the study, 408 had untreated hypertension and no history of CVD (weighted proportion: 4.7%, 95% CI 4.2–5.2), 1616 had treated hypertension and no history of CVD (17.2%, 95% CI 16.3–18.1), 679 had a history of CVD (7.6%, 95% CI 7.0–8.2), and 6230 were normotensive and with no history of CVD (70.5%, 95% CI 69.8–82.3). The proportion of untreated hypertension in males was 7.2% (6.3–8.1) and 2.4% (2.0–2.9) in females (Table 2). The same figures for treated hypertension were 17.3% (16.1–18.7) and 17.0% (15.9–18.2), respectively. The mean age of those with untreated hypertension was 57 (56–58.1) in males and 58 (56.4–58.2) in females (Table 2). The same figures for those with treated hypertension were 60.4 (59.6–61.1) and 61.9 (61.2–62.5), respectively. Men were nearly three times more likely than women to have untreated hypertension (adjusted odds ratio (AOR) when compared to treated hypertension: 2.74; 95% CI 2.10 to 3.59) (Table 3). In general, unhealthy behaviours were more associated with the untreated compared to those with treated hypertension, e.g. smokers were two and half times as likely as non-smokers to be in the untreated group (AOR 2.57; 1.85 to 3.57). Those with good–excellent self-reported health more than one and a half times as likely as those with poor–medium category self-reported health (AOR 1.73; 1.27 to 2.36). Similar results were found in those with full fat dairy preference (AOR 1.67; 1.16 to 2.42), white bread preference (AOR 1.21 to 2.18), higher alcohol consumption (AOR 1.49; 1.04 to 2.15), and living alone (AOR 1.42; 1.01 to 1.99). Strong negative associations were in the same analysis found for with being on more than five prescription drugs (AOR 0.10; 0.06 to 0.18), possession of statins (AOR 0.18; 0.12 to 0.27) or antiplatelets (AOR 0.21; 0.11 to 0.38), diabetic status (AOR 0.25; 0.14 to 0.44), and LTLI status (AOR 0.36; 0.27 to 0.48). The unadjusted and adjusted associations were higher in those with more education, higher incomes, and residence in more affluent neighbourhoods. The association with higher qualification was significantly different from no education in unadjusted analysis, but the association was weaker and non-significant in the age- and sex-adjusted analysis.

## 4 Discussion

The prevalence of untreated hypertension and no history of CVD was 7% for men, 2% for women, and 5% overall. Untreated hypertension was particularly high among the 55–64 year age group. Sex- and age group-adjusted analyses of untreated versus treated hypertension among individuals with no history of CVD found strong positive associations with male gender, smoking, self-reported good–excellent health, full fat dairy preference, white bread preference, higher alcohol consumption, and living alone. Strong negative associations were found for possessing 5 + prescription drugs, statins or antiplatelets, being diagnosed with diabetes or possessing antidiabetics, and LTLI status. Those with relatively more education, higher incomes, and residence in more affluent neighbourhoods were more likely to be in the untreated category than the treated group, although the gradients were weak and non-significant.

To our knowledge no other study has considered the group of individuals with untreated hypertension that are in effect missed by the general primary prevention CVD screening on the basis of treatment eligibility. Knott and Mindell (2011) included in a study based on Health Survey for England 2011 data anyone with hypertension regardless of whether eligible for treatment and furthermore depended on participants being able to confirm the indication of antihypertensive drugs in their possession. Their prevalence rates were higher, and also higher in men than women, although the differences between men and women were less pronounced than in this study. Appleton et al. (2012) also found higher prevalence rates in men in an Australian general population study. They did not include any treatment eligibility criteria and also found the higher rates in younger age groups.

We found an association between having untreated hypertension and living alone, which fits the growing literature on the importance of structural and functional social support/control for health outcomes (Robards et al., 2012). This finding is also consistent with studies of non-attendance to general health checks (Dryden et al., 2012), and a wide range of morbidity and mortality studies including use of antihypertensives (Kravdal and Grundy, 2014, Robards et al., 2012). The association could be studied further to investigate whether linked to non-adherence and whether amenable to e.g. additional follow-up in clinical practice.

Hypothetically, those with untreated hypertension would be expected to have similar characteristics to the groups less likely to consult GPs and less likely to attend health screening programmes. In consistency with our study English GP audit studies have so far found poorer uptake of the NHS Health Checks among younger age groups, smokers and those without co-morbidities (Artac et al., 2013a, Cochrane et al., 2013, Dalton et al., 2011, Kumar et al., 2011). One study have found poorer uptake among men (Dalton et al., 2011) and another showed poorer uptake among women (Cochrane et al., 2013). A literature review of uptake to general health checks also found worse uptake rates among smokers (Dryden et al., 2012).

Hypertension is a ‘hidden’ disease, which is more likely to be treated in patients who consult more. Treatment can for that reason become correlated with relatively poor health. This may explain why untreated hypertension – albeit non-significantly – was correlated with having more education, higher income, and residing in more affluent neighbourhoods.

Untreated compared to treated hypertension was found to be associated with higher prevalence of unhealthy behaviours. This may reflect that those being treated also having changed their risk behaviours. It is not possible to draw conclusion about this from this study.

The study highlights a group who in effect are not currently reached by the general health check programme. This is likely to be associated with the ‘hidden’ nature of hypertension; many e.g. reported their general health as good to excellent. One of the challenges facing the programme seems to be to motivate symptom-free individuals to take part.

### 4.1 Strength and limitations

General household surveys cover a broad section of the population and not only those in frequent contact with the healthcare system. It is in that way possible to capture individuals who are in effect hard to reach by general screening programmes.

Detection of hypertension in a survey will differ from how it is diagnosed in clinical practice, where it is based on both clinical assessment and a period of home monitory of blood pressure. In both clinics and survey a ‘white coat’ effect can be observed. We have reduced this impact on our findings by only using the second and third blood pressure measurements, but it is still possible that the prevalence reported here is over-estimated for this reason.

Our definition of treatment is based on being prescribed relevant antihypertension medications. Individuals who have changed their behaviours to successfully control their blood pressure (either with GP advice or by themselves) cannot be identified in this study.

The health behaviours that were included were all subjectively measured and were as such prone to recall and reporting biases. Physical activity was divided into types including walking, cycling to work and participation in moderate intensity sports. Although objectively measured physical activity has advantages, it may lack the types and context of the subjectively measured behaviours and many of the techniques are yet to be validated in epidemiological research (Atkin et al., 2012). Sedentary behaviours were not measured. Survey information was available for some on e.g. hours spent watching TV, but other studies have shown that this activity is not necessarily correlated with sedentary behaviours overall (Atkin et al., 2012). Data on other dietary habits relevant to CVD prevention were not available in this study, e.g. salt intake, fish, meat and fat preferences, total calorie intake, cooking practices, adherence to certain diets, treat food use, etc. (Ffion Lloyd-Williams, 2014, Hartley et al., 2013, Jørgensen et al., 2012, Moreira et al., 2015).

Known risk factors for screening non-attendance (Dryden et al., 2012) not studied here relate to individuals' capability, opportunity, and motivational factors (Cane et al., 2012). Qualitative studies of hypertension have from another angle highlighted the misconceived belief that hypertension is not a chronic condition, but rather an illness brought on temporarily by stress and that many stop taking antihypertensives when they feel less stressed (Marshall et al., 2012). These areas would be of interest for future research.

## 5 Conclusion

Untreated hypertension is particularly high among men and the 55–64 year olds and clustered with other risk factors for CVD such as smoking and less healthy dietary choices. Social support may also play a role as the level of untreated hypertension was higher among those living alone compared to those living with others. Notably, many reported their health as good to excellent, a fact which emphasises the importance of motivating individuals to take part in the general health checks for a ‘hidden’ or asymptomatic condition such as hypertension. The association with living alone should be studied further to investigate whether linked to non-adherence and whether amenable to e.g. additional follow-up.

## Conflicts of interest

No conflicts of interests were declared.

## Figures and Tables

**Table 1:** Characteristics of participants with no history of CVD: untreated versus treated hypertension (weighted frequencies and percentages).

	Untreated hypertension		Treated hypertension	
N	%	N	%
Gender				
Male	266	73.6	644	48.8
Female	95	26.4	675	51.2
Age groups				
40–54 years	131	36.2	304	23
55–64 years	156	43.2	466	35.3
65–74 years	74	20.5	549	41.6
Qualifications				
Higher education	114	31.7	356	27
GCSE, A-level or other	188	52.2	619	47
No qualifications	58	16.1	342	26
Net eq. household income per month				
<£1200	119	33	510	38.7
£1200–1800	120	33.3	435	33
£1800 +	122	33.7	374	28.4
Area deprivation quintiles				
Q1.Least deprived	94	26.1	310	23.5
Q2	74	20.4	266	20.2
Q3	69	19	275	20.8
Q4	66	18.3	236	17.9
Q5.Most deprived	58	16.2	231	17.6
Rural locality				
No	270	74.8	1025	77.7
Yes	91	25.2	294	22.3
Living alone				
No	283	78.5	1073	81.3
Yes	78	21.5	246	18.7
Current smoker				
No	234	64.7	1100	83.4
Yes	127	35.3	219	16.6
Alcohol consumption (relative to recommended)				
< 1.5	58	16	283	21.5
1.5 +	191	53	453	34.3
No data	112	31	583	44.2
Low physical activity				
No	92	25.5	239	18.1
Yes	269	74.5	1080	81.9
< 5 portions of fruit/veg a day				
No	77	21.3	353	26.8
Yes	284	78.7	966	73.2
White bread preference				
No	205	56.6	913	69.2
Yes	157	43.4	406	30.8
Full fat dairy preference				
No	297	82.2	1180	89.5
Yes	64	17.8	139	10.5
Obese				
No	186	51.6	656	49.7
Yes	175	48.4	663	50.3
LTLI				
No	227	62.7	489	37.1
Yes	135	37.3	830	62.9
Diabetic				
No	338	93.6	1048	79.5
Yes	23	6.4	271	20.5
Statins				
No	319	88.2	741	56.2
Yes	42	11.8	578	43.8
Antiplatelets				
No	346	95.8	1080	81.9
Yes	15	4.2	239	18.1
Antidepressants				
No	333	92.1	1170	88.7
Yes	29	7.9	149	11.3
Polypharmacy				
No	341	94.3	793	60.1
Yes	20	5.7	526	39.9
General health good–excellent				
No	93	25.7	479	36.3
Yes	268	74.3	840	63.7
Total	361	100	1319	100

**Table 2:** Untreated versus treated hypertension among individuals with no history of CVD: weighted mean age and weighted proportion.

Population	Mean age (years; (95% CI))	Proportion (95% CI)
CVD-free untreated hypertension		
Male	57.0 (56.0;58.1)	7.2% (6.3;8.1)
Female	58.0 (56.3;60.0)	2.4% (2.0;2.9)
Total	57.3 (56.4;58.2)	4.7% (4.2;5.2)

CVD-free treated hypertension		
Male	60.4 (59.6;61.1)	17.3% (16.1;18.7)
Female	61.9 (61.2;62.5)	17.0% (15.9;18.2)
Total	61.1 (60.6;61.6)	17.2% (16.3;18.1)

**Table 3:** Unadjusted and sex/age group adjusted logistic regression models of untreated versus treated hypertension among individuals with no history of CVD.

Untreated vs treated CVD-free hypertension		Unadjusted	P-values	Adjusted^c^	P-values
OR (95% CI)	AOR (95% CI)
Gender	Female	Ref		Ref	
Male	2.92 (2.24–3.80)	< .01	2.74 (2.10–3.59)	< .01
Age group	40–54 years	Ref		Ref	
55–64 years	0.78 (0.55–1.10)	.16	0.80 (0.56–1.13)	.21
65–74 years	0.31 (0.22–0.46)	< .01	0.34 (0.23–0.50)	< .01
Education	Higher education	Ref		Ref	
GCSE, A-level	0.95 (0.69–1.31)	.75	1.03 (0.74–1.43)	.86
No quals.	0.53 (0.35–0.80)	< .01	0.78 (0.50–1.21)	.27
Household income^a^	<£1200	Ref		Ref	
£1200–1800	1.18 (0.85–1.64)	.31	1.07 (0.76–1.50)	.71
£1800 +	1.39 (0.97–1.98)	.07	1.13 (0.78–1.64)	.53
Area deprivation^b^	Q1.Least deprived	Ref		Ref	
Q2.	0.91 (0.61–1.37)	.66	0.96 (0.63–1.48)	.86
Q3.	0.82 (0.53–1.29)	.39	0.84 (0.53–1.32)	.44
Q4.	0.92 (0.62–1.38)	.70	0.87 (0.57–1.31)	.50
Q5.Most deprived	0.83 (0.53–1.30)	.42	0.73 (0.46–1.16)	.18
Rural locality	No	Ref		Ref	
Yes	1.17 (0.87–1.58)	.29	1.34 (0.98–1.83)	.06
Living alone	No	Ref		Ref	
Yes	1.20 (0.86–1.66)	.28	1.42 (1.01–1.99)	.04
Current smoker	No	Ref		Ref	
Yes	2.74 (1.97–3.81)	< .01	2.57 (1.85–3.57)	< .01
Alcohol	< 1.5 × recom'd	Ref		Ref	
1.5 +	2.06 (1.45–2.93)	< .01	1.49 (1.04–2.15)	.03
No data	0.94 (0.65–1.35)	.73	0.90 (0.61–1.33)	.60
Low physical activity	No	Ref		Ref	
Yes	0.65 (0.49–0.86)	< .01	0.75 (0.55–1.03)	.07
< 5 fruit/veg per day	No	Ref		Ref	
Yes	1.35 (1.00–1.82)	.05	1.05 (0.77–1.43)	.76
White bread	No	Ref		Ref	
Yes	1.72 (1.30–2.28)	< .01	1.63 (1.21–2.18)	< .01
Full fat dairy	No	Ref		Ref	
Yes	1.84 (1.28–2.66)	< .01	1.67 (1.16–2.42)	< .01
Obese	No	Ref		Ref	
Yes	0.93 (0.71–1.22)	.59	0.87 (0.65–1.15)	.32
Long-term limiting illness	No	Ref		Ref	
Yes	0.35 (0.27–0.46)	< .01	0.36 (0.27–0.48)	< .01
Diabetic	No	Ref		Ref	
Yes	0.26 (0.15–0.45)	< .01	0.25 (0.14–0.44)	< .01
Statins	No	Ref		Ref	
Yes	0.17 (0.11–0.26)	< .01	0.18 (0.12–0.27)	< .01
Antiplatelets	No	Ref		Ref	
Yes	0.20 (0.11–0.36)	< .01	0.21 (0.11–0.38)	< .01
Antidepressants	No	Ref		Ref	
Yes	0.68 (0.44–1.03)	.07	0.79 (0.52–1.20)	.27
Polypharmacy	No	Ref		Ref	
Yes	0.09 (0.05–0.16)	< .01	0.10 (0.06–0.18)	< .01
Gen. health good–excellent	No	Ref		Ref	
Yes	1.65 (1.21–2.25)	< .01	1.73 (1.27–2.36)	< .01

a Net equivalised household income.

b Townsend area deprivation quintiles at Census 2011 lower layer super output area level.

c Adjusted for sex and age group.
